# Adhesion extent data of repaired rabbit Achilles tendons three weeks post-surgery and characterization data of different implant materials used for these surgeries

**DOI:** 10.1016/j.dib.2024.111069

**Published:** 2024-10-23

**Authors:** Iris Miescher, Nicola Schaffner, Julia Rieber, Gabriella Meier Buergisser, Esteban Ongini, Yao Yang, Athanasios Milionis, Viola Vogel, Jess G. Snedeker, Maurizio Calcagni, Johanna Buschmann

**Affiliations:** a Division of Plastic Surgery and Hand Surgery, University Hospital Zurich, Sternwartstrasse 14, Zurich 8091, Switzerland; b University Clinic Balgrist, Orthopaedic Biomechanics, Forchstrasse 340, Zurich 8008, Switzerland; c Department of Health Sciences & Technology & Department of Materials, Schmelzbergstrasse 9, LFO, Zürich 8092, Switzerland; d Laboratory of Thermodynamics in Emerging Technologies, Department of Mechanical and Process Engineering, ETH Zürich, Zürich 8092, Switzerland; e Laboratory of Applied Mechanobiology, and Department of Health Sciences and Technology, ETH Zurich, Institute of Translational Medicine, Zurich 8093, Switzerland

**Keywords:** Hyaluronic acid (HA), Polyethylene oxide, Tendon repair, Tenocytes, Electrospinning

## Abstract

As one major problem after tendon rupture repair, surgeons are confronted with fibrotic adhesion formation of the healing tendon to the surrounding tissue. Although early active motion is recommended during rehabilitation, adhesions may lead to joint stiffness and a restricted range of motion. One viable option to counteract adhesion formation is to add a thin elastic tube that is placed over the conventionally sutured repair site. Such a tube reduces adhesion formation because it acts as a physical barrier. Additionally, such barriers can be optimized by adding a biolubricant.

We here present adhesion data of rabbit Achilles tendons that were fully transsected, repaired with a 4-strand suture and received a) no implant; b) an electrospun DegraPol tube and c) a bi-layered tube with one electrospun DegraPol layer and one high molecular weight hyaluronic acid (HA)/polyethylene oxide (PEO) electrospun layer. Based on Picrosirius red stained tendon cross-sections three weeks post-operation, the percentage of adhesion data is presented. Moreover, mechanical data of the implant materials are presented as a further dataset, with the following readouts: fracture strain [%], ultimate tensile stress [MPa] and Young's modulus [MPa]. They are presented in axial and transverse stretching directions, respectively.

The adhesion data can be reused for comparison to other implant materials, drugs or anti-adhesive strategies that are applied in similar pre-clinical models like the rabbit Achilles tendon model. The mechanical data of the implant materials offer the possibility to compare electrospun meshes based on other polymers to the materials used here or for computational models of such materials.

Specifications Table

**Table:** 

Subject	Surfaces, coatings, films
Specific subject area	Adhesion extent data for different implant materials applied around sutured rabbit Achilles tendons 3 weeks post-operation
Data format	Raw, Analyzed
Type of data	.xlsx files (data sets with group labels and numbers)
Data collection	Light microscope (Leica EZ4D microscope, Switzerland) to acquire images of histological sections; assessment of adhesion extent with synedra view software (version 22.0.0.12) by measuring the length of the contact region and dividing it by the length of the total perimeter of the tendon. Uniaxial tensile testing machine (Zwick Z010, 20 kN load-cell, testXpert III; Zwick/Roell, Ulm, Germany) to acquire stress-strain curves of the electrospun implant materials. Calculation of the parameters was performed with MATLAB (Release 2021a, The MathWorks, Inc., Natick, MA, USA). The thickness of the electrospun meshes was measured using microscopic images of a × 4 objective (AE2000, Motic, TX USA).
Data source location	Adhesion extent data: University Hospital Zurich, Switzerland Mechanical data of implants: Balgrist University Hospital, Switzerland
Data accessibility	**Repository name**: Medeley Data (Mendeley Data) **Data identification number**: 10.17632/zr7rvp6str.2 Direct **URL** to data: Hyaluronic acid/PEO electrospun tube reduces tendon adhesion to levels comparable to native tendons – an in vitro and in vivo study - Mendeley Data
Related research article	Iris Miescher, Nicola Schaffner, Julia Rieber, Gabriella Meier Buergisser, Esteban Ongini, Yao Yang, Athanasios Milionis, Viola Vogel, Jess G. Snedeker, Maurizio Calcagni and Johanna Buschmann Hyaluronic acid/PEO electrospun tube reduces tendon adhesion to levels comparable to native tendons – an in vitro and in vivo study, [1]. Link: Hyaluronic acid/PEO electrospun tube reduces tendon adhesion to levels comparable to native tendons - An in vitro and in vivo study - PubMed (nih.gov)

## 1 Value of the Data

• The adhesion extent dataset is useful in tendon research, as they reflect variation in adhesion formation under the same surgical treatment.
• The adhesion extent dataset can be used for comparison with other (pre-) clinical anti-adhesion strategies in general or with other artificial barriers in specific.
• The mechanical data set of the implants provides information on anisotropy of electrospun polymer fiber meshes. They can be utilized as reference mechanical data for material scientists using other polymers.
• The mechanical data can be used in the development of new computational models describing mechanics of electrospun polymer fiber meshes.

## 2 Background

In plastic surgery and traumatology, tendon repair is confronted with two major problems. Scar formation during healing [2] may lead to mechanically inferior tissue and fibrotic adhesion formation to the surrounding tissue may results in restricted range of motion [3]. To address the problem of adhesion formation, we developed an implant in form of an electrospun fibre mesh that should act as an artificial physical barrier when pulled over a conventionally sutured tendon [4]. We have therefore developed a non-bioactive version with pure DegraPol polymer and a bioactive version of such a tube, realized as bi-layer electrospun mesh having one pure DegraPol layer and one hyaluronic acid/PEO blend layer [1]. We tested them in the rabbit Achilles tendon full transection model [5,6] and provide datasets on adhesion extent to the surrounding tissue evaluated three weeks post-surgery. These data offer surgeons insight into biological variability of surgical treatment and reference values for other anti-adhesion strategies evaluated in pre-clinical models similar to the full transection rabbit Achilles tendon. Furthermore, we characterized the electrospun tubes by mechanically testing them, using an uniaxial tensile testing machine [1]. Such data offer other researchers reference values for their own electrospun implant materials and may furthermore be utilized for computational models.

## 3 Data Description

The data are stored as Microsoft Excel (Microsoft Corporation, Redmond, WA, USA) files (.xlsx files) in the Mendeley Data repository service Hyaluronic acid/PEO electrospun tube reduces tendon adhesion to levels comparable to native tendons – an in vitro and in vivo study - Mendeley Data.

1. **Adhesion Data**
  The data for adhesion formation expressed as percentage of adherent tissue based on whole circumference is stored at Hyaluronic acid/PEO electrospun tube reduces tendon adhesion to levels comparable to native tendons – an in vitro and in vivo study - Mendeley Data.

**File 1** InVivo_Adhesion.xlsx

This excel file is open access published in Mendeley Data (Hyaluronic acid/PEO electrospun tube reduces tendon adhesion to levels comparable to native tendons – an in vitro and in vivo study - Mendeley Data) and contains the group labels in column A and the percentage of adhesion as numbers in column B, where for example 0.51 means 51 % of adhesion formation. The key for the abbreviations (labels) in column A is: NT = not treated tendons, no operation, native contralateral sides; 4-strand = 4-strand Becker sutured tendons; DP = 4-strand Becker sutured tendons with a pure DegraPol tube; DP/HA/PEO = 4-strand Becker sutured tendons with a bi-layered tube consisting of one layer of pure DegraPol and one layer of a HA/PEO blend 1:1.

2. **Mechanical Data**
  **File 2** MechanicalTests.xlsx

This excel file is open access published in Mendeley Data (Hyaluronic acid/PEO electrospun tube reduces tendon adhesion to levels comparable to native tendons – an in vitro and in vivo study - Mendeley Data) and contains four sheets, denoted as Axial, Transverse, Ring and Wall diameter, respectively. Axial, Transvers and Ring sheets have the same order of columns with the same parameters. Columns A-D refer to the HA/PEO blend in a weight/weight ratio of 1:1, indicated as HA/PEO 1:1. Columns F-I refer to the HA/PEO blend in a weight/weight ratio of 1:4, indicated as HA/PEO 1:4. Columns K-N refer to the pure Degrapol mesh, indicated as DP. For each of the three materials, i.e. HA/PEO 1:1, HA/PEO 1:4, and DP, the first columns (A, F and K) denote the labeling of the tube, the second column (B, G, L) denote the values obtained for the ultimate tensile stress in MPa, the third column (C, H, M) gives the values obtained for the fracture strain in %, and the fourth column denotes values obtained for the Young's modulus in MPa.

The last sheet (Wall diameter) gives the thickness of the wall of each specimen in mm. In column A, the three materials and the labels of the tubes are shown; in column B the thickness is given, in column C the average (mean) is given for each material and in column D the standard deviation is given for each material.

3. **Fiber thickness in electrospun fiber meshes**
  File 3 Fiberthickness_SEM.xlsx

This excel file is open access published in Mendeley Data (Hyaluronic acid/PEO electrospun tube reduces tendon adhesion to levels comparable to native tendons – an in vitro and in vivo study - Mendeley Data) and contains thirteen sheets, denoted as Tube 02, Tube 03, Tube 05, Tube 06, Tube 04, Tube 10, Tube 11, DP Mean & SD, DP sortiert, 1_1 Mean & SD, 1_1 sortiert, 1_4 Mean & SD, 1_4 sortiert. The sheets starting with Tube are organized the same, with the following Key. Column B “Well Nummer” means well number and shows in which well the tube lay in the corresponding 6-well plate. Column C “Bildnummer” means the number of the image where the fiber thickness was determined. Column D “Magnification” indicates at which magnification the fiber thickness was assessed. Column D “Point of view (SS, IS, AS, BS)” means which parameter was determined, with SS = Thickness of the section; IS = fiber thickness of inner surface of the tube; AS = fiber thickness of outer surface of the tube; and BS = thickness of the layer. Column F “Länge (in um)” means length in mm. Column G “Mittelwert (in um)» means average in mm. Column H « Standarabweichung (in um)» means standard deviation in mm. Column K on line 2 says “Gesammt-Schichtdicke über alle Wells:” which denotes the average of the thickness of all sections. Column K on line 3 says “Gesammt-STAB-Schichtdicke:” denoting the standard deviation of the average of the thickness of all sections. Column K on line 4 says “Gesammt-Faserdicke (Aussenseite) über alle Wells:» which denotes the average of the fiber thickness of outer surface of the tube. Column K on line 5 says “Gesammt-STAB-Faserdicke (Aussenseite):” which means the standard deviation of the fiber thickness of outer surface of the tube”. Column M line for says “Gesammt-Faserdicke (Innenseite) über alle Wells:” which denotes the average of the fiber thickness of inner surface of the tube. Column M on line 5 says “Gesammt-STAB-Faserdicke (Innenseite):” which means the standard deviation of the fiber thickness of inner surface of the tube”.

Then the sheet “DP Mean & SD” shows in column B all raw data for the fiber thickness for the DegraPol tubes’ outer surfaces. In column C then there is mean and SD (standard deviation) of these raw data. The same is true for the sheet “1_1 Mean & SD”, however, it is for the electrospun HA:PEO = 1:1 tubes. The same is also true for the sheet” 1_4 Mean & SD”, however, it is for the electrospun HA:PEO = 1:1 tubes. As for the remaining sheets, the sheet “DP sortiert” shows the same raw data than the sheet “DP Mean & SD”, but in column A, the raw data are ordered in ascending numbers. In column C, there are the ranges of fiber thicknesses. In column D then, there are the amount of fibers that fall within a specific range. In column E and F, the numbers denote the fracture of the amount of fibers falling within a specific range, in %. The same is true for “1_1 sortiert” and for “1_4 sortiert”, just for the other materials: HA:PEO = 1:1 tubes and HA:PEO = 1:4 tubes, respectively.

4. **Pore size in electrospun fiber meshes**
  File 4 Poresize_SEM.xlsx

This excel file is open access published in Mendeley Data (Hyaluronic acid/PEO electrospun tube reduces tendon adhesion to levels comparable to native tendons – an in vitro and in vivo study - Mendeley Data) and contains six sheets, denoted as HA_PEO (1_1); HA_PEO (1_1) sorted; HA_PEO (1_4); HA_PEO (1_4) sorted; DP and DP sorted, respectively. The first, the third and the fifth sheet are organized the same; while the second, the fourth and the sixth sheet are also organized the same. All sheets without the name part “sorted” are organized as follows: Column A shows the name of the tube, column B gives the position within the picture, column C is referring to a specific pore, giving the pore a (random) number, column D refers to a specific SEM image at a magnification of 700x, column E indicates a starting and column F an end position (arbitrary numbers) within the specific SEM image, column G shows the pore size in mm, column I line 1 indicates the mean value of all pores, while column I line 2 (STD) shows the standard deviation of the mean value of all pores sizes (both in mm). All sheets with the name part “sorted” are organized the same: In column A, in ascending order all raw data of pore sizes are listed, in column C there are ranges of pore sizes, in column D is the number of pores measured that belong to a specific range of pore size (and at the bottom the sum of all numbers), in column E and F the percentage of the number of pore sizes belonging to a certain range is given.

5. **Fourier Transformed Infrared Spectroscopy**
  **File 5** FTIR.xlsx

This excel file is open access published in Mendeley Data (Hyaluronic acid/PEO electrospun tube reduces tendon adhesion to levels comparable to native tendons – an in vitro and in vivo study - Mendeley Data) and contains four sheets, denoted as Normalize of DP_HA; Berechnung norm. Mittelwerte; Ratio; and Powder, respectively. For the sheet Normalize of DP_HA, in column A the wavenumber of the FTIR spectrum is given in cm^-1^. In all other columns line 1 shows the name of the specific sample; in the lines below line 1 there are the transmission values corresponding to a specific wavenumber of column A. DP15 means DegraPol; HA11 means HA:PEO in a ratio of 1:1; HA14 means HA:PEO in a ratio of 1:4. For the sheet Berechnung norm. Mittelwerte, which means calculation of normalized average values, line 1 gives the name of the specific sample or (highlighted in yellow) denotes the average value (column M, Z, and AM). In the sheet Ratio, the ratio of C-O to C=O transmission was calculated based on the transmission values from sheet Berechnung norm. Mittelwerte. Here, column A shows the specific name of the tube, column B shows the values for the C-O (1153-1157), where 1153–1157 cm^-1^ is the range of wavenumbers where the value for transmission was taken from. Column C shows the transmission values for the C=O bond at 1722 cm^-1^. Column D finally shows the calculated ratio of the transmission values for C=O/C-O. The sheet Powder is organized in the same way as the sheet Normalize of DP_HA, however, some samples are not tubes as in the sheet Normalize of DP_HA, but corresponding powders were assessed. In line 1, the specific name of the samples can be seen; for example HA dried means dry hyaluronic acid powder; PEG dried means dry poly ethylene glycol powder (35000 g/mol); PEG powder means poly ethylene glycol powder that was not dried but taken as received from the manufacturer; PEG spun means poly ethylene glycol that was electrospun; PEO dried means dry poly ethylene oxide (600000 g/mol).

6. **Water contact angle on electrospun fiber meshes**
  **File 6** FTIR.xlsx

This excel file is open access published in Mendeley Data (Hyaluronic acid/PEO electrospun tube reduces tendon adhesion to levels comparable to native tendons – an in vitro and in vivo study - Mendeley Data) and contains two sheets, denoted as Static 2022-06.03.2023; and Dynamic 06.03.2023 (Iris), respectively. In the sheet named Static 2022-06.03.2023, the static water contact angle was assessed and values are given in column B in (°), while column A gives the name of the specific sample, with the following key: DP 04 L (March) = DegraPol tube number 04 left angle of the water droplet, assessed in March 2023; DP 04 R (March) = DegraPol tube number 04 right angle of the water droplet, assessed in March 2023; DP Mai L (2022) = DegraPol tube left angle of the water droplet, assessed in May 2022. Furthermore, columns D and E give the means and the standard deviations (SD) for the DegraPol tube water contact angle values of column B. Columns G-K are organized the same as columns A-E, but here the material is HA/PEO 1:1. Column M-Q are organized the same as columns A-E, however, here the material is HA/PEO 1:4. In the sheet named Dynamic 06.03.2023 (Iris), the dynamic water angles as assessed on March 7th, 2023, are shown. In lines 3, 10, 20, 27, 36 and 43, the name of the specific sample that was tested is given. Key: DP04 advancing = DegraPol tube 04 advancing water contact angle value; HA 1:1 09 receding = HA/PEO 1:1 tube number 09 receding water contact angle value. In line 5 and others, Rechts means right and refers to the right water contact angle; Links means left and refers to the left water contact angle; Histeresis means hysteresis and gives the difference of the advancing and the receding water contact angle (°).

7. **Differential Scanning Calorimetry of electrospun fiber meshes**
  **File** DSC_2.HeatingCycle.xlsx

This excel file is open access published in Mendeley Data (Hyaluronic acid/PEO electrospun tube reduces tendon adhesion to levels comparable to native tendons – an in vitro and in vivo study - Mendeley Data) and contains six sheets, denoted as DP_03; DP_04; DP_05; HA11_05; HA11_09; HA11_14; HA14_10; HA14_15; and HA14_16. All sheets are equally organized, but do refer to different samples, with DP_03 = DegraPol tube 03; HA11_05 = HA/PEO 1:1 tube number 05 or HA14_10 = HA/PEO 1:4 tube number 10. In column A the time in seconds (s) is given; in column B the temperature is given in degree Celsius (°C) and in column C the normalized heat flow in watt per gram of material (W/g) is shown. In column E and F, the derived values for GT°C = glass transition temperature in°C, the Tm°C DP = melting temperature in°C for DP = DegraPol, and the corresponding phase transition enthalpy denoted as “Enthalpie J/g DP” in Joule/gram of material DP are shown.

### 3.1 Materials

For electrospinning DegraPol (DP), a biodegradable polyester urethane block copolymer, was kindly provided by Ab Medica, Italy. High molecular weight hyaloronic acid (HA, 1.01 – 1.8 MDa) was ordered from Lifecore Biomedical (Lifecore Biomedical #HA 15M, Chaska, USA). Chloroform (#132950), 1,1,1,3,3,3-hexa fluoro-2-propanol (HFP) (#105228), polyethylene glycol (PEG) (in average 35’000 g/mol) (#81310) and polyethylene oxide (PEO) (in average 600’0000 g/mol) (#182028) were purchased from Sigma-Aldrich (Buchs, Switzerland). Solutions for electrospinning were filled in a 5 mL glass syringe (Huberlab, #3.7102.33, Aesch, Switzerland).

For cell culture, gentamycine (# L0011), Ham's F12 (# L0135-500) and fetal bovine serum (FBS) (# S1830-500) were bought from Biowest (Nuaillé, France) and amphotericin B (# P06-01100) from Pan Biotech (Aidenbach, Germany). Penicillin/streptomycin (# 15140122) and GlutaMAX™ (# 35050038) were delivered from ThermoFisher scientific (Basel, Switzerland) and phosphate buffered saline (PBS) (# D8537) from Sigma-Aldrich (Merck, Buchs, Switzerland). Cells were cultured in tissue culture plates from Corning (PrimariaTM, Corning, New York, NY, USA) and alamarBlue™ cell viability assay (# DAL1100) from Invitrogen (ThermoFisher Scientific, Basel, Switzerland) was carried out in 12-well plates from Sigma-Aldrich (Merck, Buchs, Switzerland, # 665180) and 96-well plates (# 92096) from Techno Plastic Products AG (TPP) (Trasadingen, Switzerland).

For immunocytochemical staining an Autostainer Link48 (DAKO, Basel, Switzerland) was used. Target retrieval solution (# GV80411) and washing buffer (# K800721) were ordered from DAKO (DAKO, Baar, Switzerland). Hydrogen peroxide H_2_O_2_ was bought from Sigma-Aldrich (Merck, Buchs, Switzerland, # 1.07209.0250) and Pertex from HistoLab (Biosystems, Muttenz, Switzerland #00801-EX) was used to fix coverslips. For Collagen I staining a goat polyclonal primary antibody was bought from abcam (abcam, Cambridge, UK, # ab24821; 1:100 dilution), horse anti-goat IgG antibody (H+L) peroxidase was ordered from Biozol (Biozol, Eching, Germany, # VEC-PI-9500) and horse serum from AdipoGen (AdipoGen, Liestal, Switzerland, # VC-S-2000) was used for blocking. For Fibronectin staining a mouse monoclonal primary antibody was purchased from Sigma-Aldrich (Sigma-Aldrich, Merck, Buchs, Switzerland, # F0791; 1:200 dilution) and for α-SMA staining a mouse monoclonal primary antibody from DAKO was applied (Agilent Technologies, Basel, Switzerland, # IR611; 1:2 dilution). For Fibronectin and α-SMA staining a peroxidase labelled polymer conjugated to goat anti-mouse immunoglobulins from DAKO was used (Agilent Technologies, Basel, Switzerland, # K4001) and samples were blocked with goat serum (AdipoGen, Liestal, Switzerland, # VC-S-1000).

To chemically dry scaffolds seeded with tenocytes for SEM, 1,1,1,3,3,3-hexamethyldisilazan (HMDS) (#3840.3) from Carl Roth AG (Arlesheim, Switzerland) was used.

### 3.2 Methods

#### 3.2.1 Solution Preparation for Electrospinning

One day before electrospinning, a 12 wt % DP polymer solution was prepared by dissolving DP in a mixture of chloroform/hexafluoropropanole (HFP) (80:20 wt/wt) at 22 °C. At 1:1 and 1:4 weight ratios, HA/PEO solutions (2 wt %) were prepared and dissolved in Milli-Q water, under stirring at 500 rpm for two days at 22 °C. PEG (30 wt %) was dissolved in CHCl_3_ at 22 °C and used for electrospinning the following day.

#### 3.2.2 Electrospinning

Using an in-house assembled electrospinning device consisting of a DC high voltage supply (Glassman High Voltage Inc., High Bridge, NJ, US), a spinning head with a blunt end and a stainless steel tube (1 mm inner diameter and 0.3 mm wall thickness, Angst & Pfister AG, Zürich, Switzerland), electrospinning was performed. The electrospinning head was connected via a Teflon hose with a syringe pump (SP210cZ, WPI, Germany). All solutions were filled into 5 mL glass syringes and the electrospun fibers were collected on a metal rod (6 mm diameter and 450˗550 mm length) fixated to a rotary motor (Euro Star B rotary motor, IKA Labortechnik). The rotation speed was 500 rpm. Flow rate was 1 mL/h for all solutions. 12.5 kV and a working distance of 18.5 cm were used for PEG and DP solutions, while layers containing HA/PEO were produced with 20 kV and a working distance of 14 cm. The electrospinning was performed at temperatures in the range of 22˗25 °C with an air humidity between 21 and 35 % for PEG and DP, while the humidity was kept in the range of 21–26 % for HA/PEO solutions. At higher humidities, we found that the HA/PEO solutions were not well spinnable. The first layer of all tubes consisted of a thin PEG layer in order to enable and easy detachment of the tube from the metal rod utilizing 50 % ethanol. The Teflon hose was rinsed with CHCl_3_ and dried with air between using different solutions in order not to contaminate one particular material with another material. For the in vivo experiment, a first layer of HA/PEO (either 1:1 or 1:4) was electrospun followed by a pure DP layer. Tubes were flipped before implantation, to have the rough surface facing the tendon and the smooth surface facing the surrounding tissue.

#### 3.2.3 Fiber Thickness and Pore Size

To assess scanning electron microsopy (SEM) images, electrospun scaffolds were mounted on metal stubs with conducting double-sided tape and were sputtered with 10 nm platinum (safematic CCU-010, Zizers, Switzerland). Three samples (n = 3) were analyzed for each material using SEM (Zeiss Gemini 450, Feldbach, Switzerland) with an accelerating voltage of 5 kV utilizing the secondary electron detector. Three microscopic fields per scaffold were captured at 700 × magnification and pictures were analyzed with ImageJ (1.53e/Java 1.8.0_172 (64-bit)). Specifically, the fiber thickness was assessed by drawing a diagonal line on the image and the thickness of fibers crossing the line was determined. As for the determination of the pore sizes, two diagonals were drawn and the pores on the surface of both diagonals were analyzed.

#### 3.2.4 Fourier Transformed Infrared Spectroscopy

We used a Varian 640 Fourier Transform Infrared Spectrometer (FTIR) equipped with a Golden Gate-diamond ATR with temperature control to assess FTIR spectra. 64 scans were averaged, all of them collected in a wavenumber range of 600–4000 cm^−1^ and at a resolution of 4 cm^−1^. Four scaffolds per material (n = 4) were used and three regions of interest were scanned. In addition, normalization to the C=O peak at 1722 cm^−1^ was performed using GraphPad Prism 9 for Windows (GraphPad Software, San Diego, California USA). The ratio between the C–O peak in the wavenumber range of 1153–1157 cm^-1^ and the C=O peak at 1722 cm^−1^ was calculated for comparison of the different materials. Corresponding bonds were assigned referring to the peaks in the IR-spectrum table (Merck KGaA, Darmstadt, Germany).

#### 3.2.5 Water Contact Angle (WCA)

Static and dynamic WCAs on the surface of different scaffolds were determined utilizing a video-assisted optical contact angle instrument (OCA 35 Dataphysics, Germany). To assess the static WCA, a drop of 5 µl Milli-Q water was placed on the scaffold surface. The angles on both drop sides (left and right, respectively) were measured for three scaffolds per material (n = 3) and measurements were repeated 5˗10 times per scaffold and averaged. The calculated angle mean was defined as the static WCA.

To assess the dynamic WCA, the advancing and receding WCAs were measured and hysteresis was calculated as the difference between the advancing WCA and the receding WCA. To determine the advancing WCA, an initial drop of 5 µl Milli-Q water was positioned on the scaffold and water was continuously added. Angles were measured when the drop baseline on the scaffold fitfully increased. The receding angle was measured in the same way, however, instead of adding the water like in the advancing case, here for the receding angle, we removed the water continuously from the initial 5 µl drop of water. A video of the process was taken to measure the contact angles. The left and right angles were measured using ImageJ (1.53e/Java 1.8.0_172 (64-bit)) and averages were calculated. Three samples were used per material (n = 3) and three measurements were carried out per sample.

#### 3.2.6 Differential Scanning Calorimetry (DSC)

Thermal analysis of the samples was carried out using a differential scanning calorimeter, DSC2500 (TA Instruments). Four scaffolds per material (n = 4) weighing between 3 and 15 mg were measured. With a heating and cooling rate of 10 °C/min, two heating cycles with an intermediate cooling cycle were executed, in the temperature range of -90 °C up to 170 °C. Only values of the second heating cycle were considered to compare the different samples. The glass transition point and the phase transition enthalpy were calculated with the software provided by the DSC machine (TA Instruments TRIOS v5.1.1.46527).

#### 3.2.7 Mechanical Tests

**Fig. 1:**
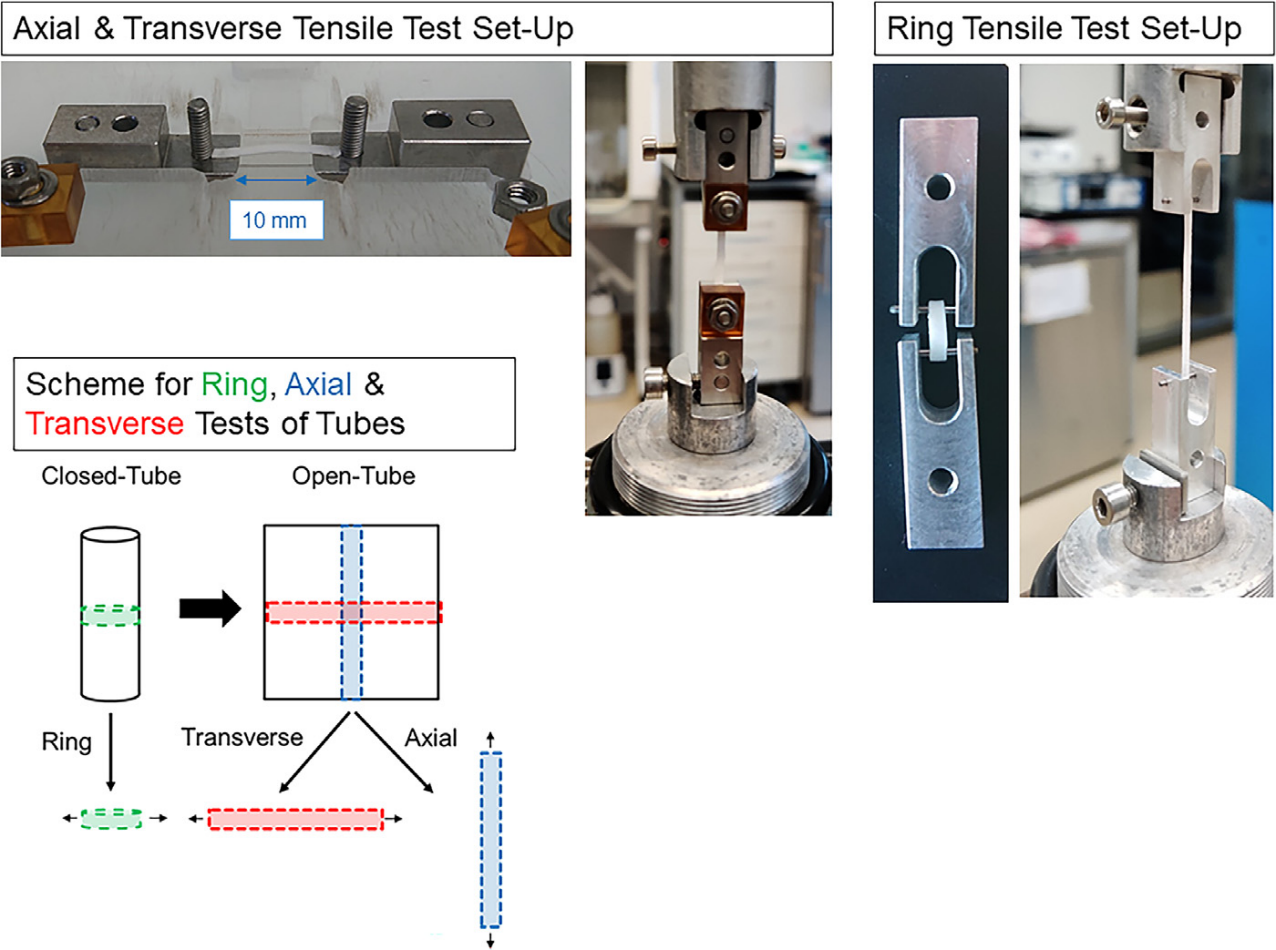
Tensile testing set-up. The electrospun tubes were mechanically characterized in axial direction as well as in transverse directions (blue). Transverse assessments were performed open (denoted as transverse, red) or as ring pieces (denoted as ring, green). Tensile testing was executed for mere polymer DegraPol tubes and for both HA/PEO tubes (in a 1:1 and a 1:4 ratio blend).

To measure the stress-strain curve of the different materials a uniaxial load test machine (Zwick Z010, 20 kN load-cell, testXpert III; Zwick/Roell, Ulm, Germany) was used to measure the stress-strain curve of the different materials. A strain rate of 10 mm/min until failure was applied for all specimens. To perform axial and transverse measurements, samples were cut into rectangular pieces with an area of 2 mm × 18 mm. They were clamped to an initial gauge length of 10 mm (Fig. 1). For ring measurements, 2 mm specimens were cut and clamped to a gauge length of 8 mm. Ultimate tensile stress (UTS), fracture strain and the Young's modulus were determined as the peak stress to failure, strain at failure, and the slope of a linear fit of the stress-stain curve up to 20 % strain, respectively. MATLAB (Release 2021a, The MathWorks, Inc., Natick, MA, USA) was utilized for the calculation of mechanical parameters. Force divided by specimen cross sectional area was defined as stress; and strain as the percent change in length from the initial gauge length. For the HA/PEO containing tubes, 3 tubes per material (n=3) were used and 6 measurements per tube were performed. For the pure DP tubes, 2 samples (n=2) with 6 repetitions were measured.

#### 3.2.8 In Vivo Implantation

**Fig. 2:**
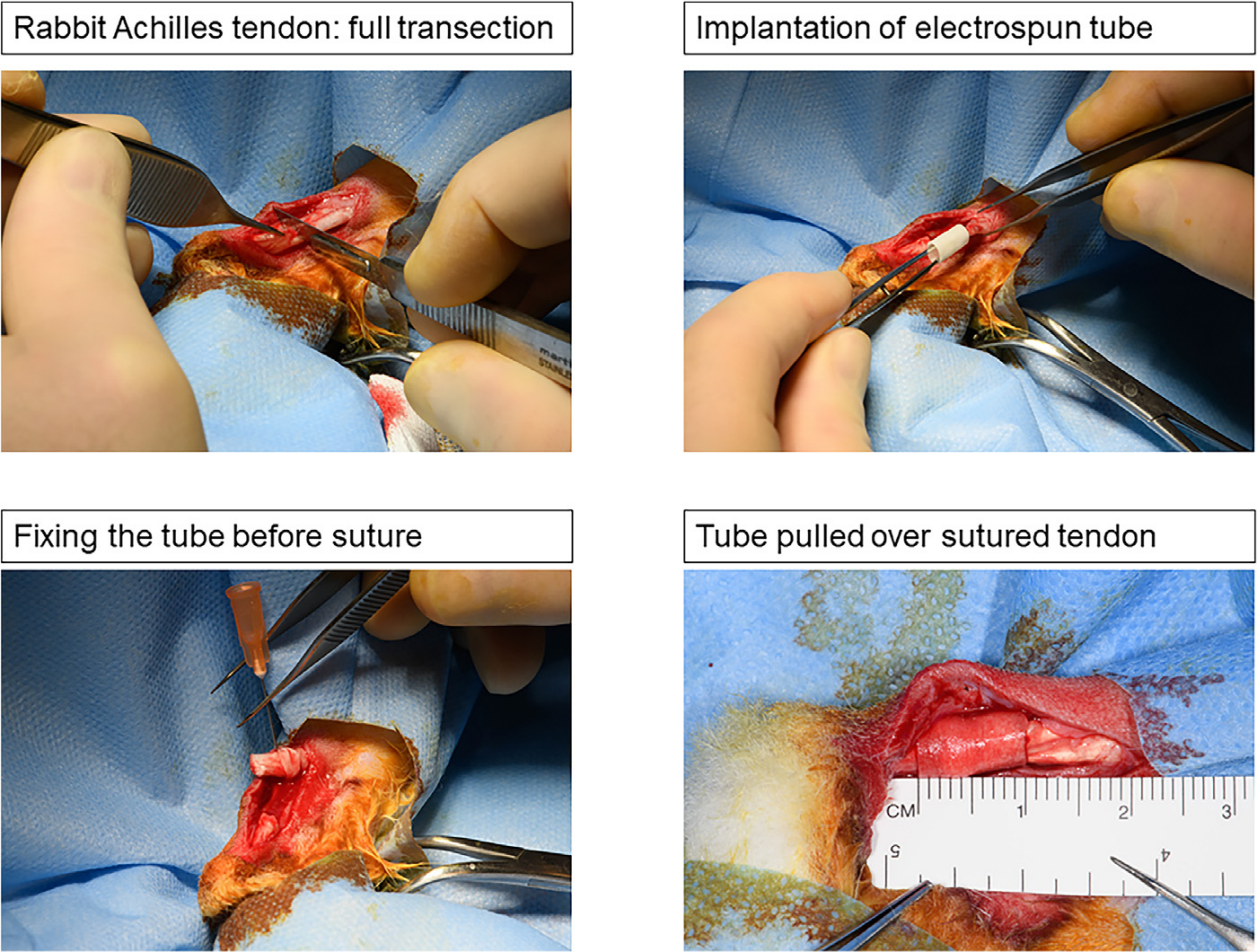
Tube application in the full transection rabbit Achilles tendon model. First, the Achilles tendon is cut by a scalpel. Then, the tube is pulled over one stump (here proximal). Then, the tube is fixed. After that, the tendon is sutured. Finally, the tube is pulled over the freshly sutured site. The tube has a length of 1 cm. Tubes of HA/PEO were implanted in a blend of 1:1.

Three female New Zealand White rabbits were used for the implantation of HA/PEO tubes in a ratio 1:1. The rabbits were aged 12–16 weeks, specific pathogen free (SPF; from Charles River, Research Models and Services, Germany). The animals were acclimatized to their environment for 2 weeks before surgery, maintained and fed as previously described [7]. Ethical approval for the experiments was achieved from the veterinary office of Zurich, Switzerland (reference numbers ZH 080/2021; 33530). Surgery was performed on one hind leg while the counter hind leg was not treated and served as control. The full transection of the Achilles tendon 2 cm above the calcaneus, followed by a 4 -strand Becker suture was carried out as described previously [7]. Plasma sterilization was used to sterilize the tubes using H_2_O_2_. Before implantation, the tubes were flipped over the wound so that the smooth HA containing layer was facing the circumadjacent tissue (Fig. 2). Then the wound was closed with a running suture with a USP 6.0 polypropylene thread and a well-padded cast was applied with an angle of 180° at the ankle to avoid stress on the Achilles tendon. Each rabbit received a Durogesic Matrix patch after surgery (Janssen-Cilag AG, Switzerland) with 4.2 mg Fentanyl per patch to provide sustained analgesia for approximately 72 h; specifically using a 25 µg/h Fentanyl patch. Three weeks post-surgery, the rabbits were euthanized in deep anaesthesia (100 mg/kg Ketamine and 4 mg/kg Xylazine) with 80 mg/kg Pentobarbital (Esconarkon ad us. vet., Switzerland) and the tendons were harvested. All extracted tendons were instantly frozen and stored at -20 °C in a gauze saturated with a 0.9 % NaCl-solution.

#### 3.2.9 Histological Analysis of Repaired Tendons

**Fig. 3:**
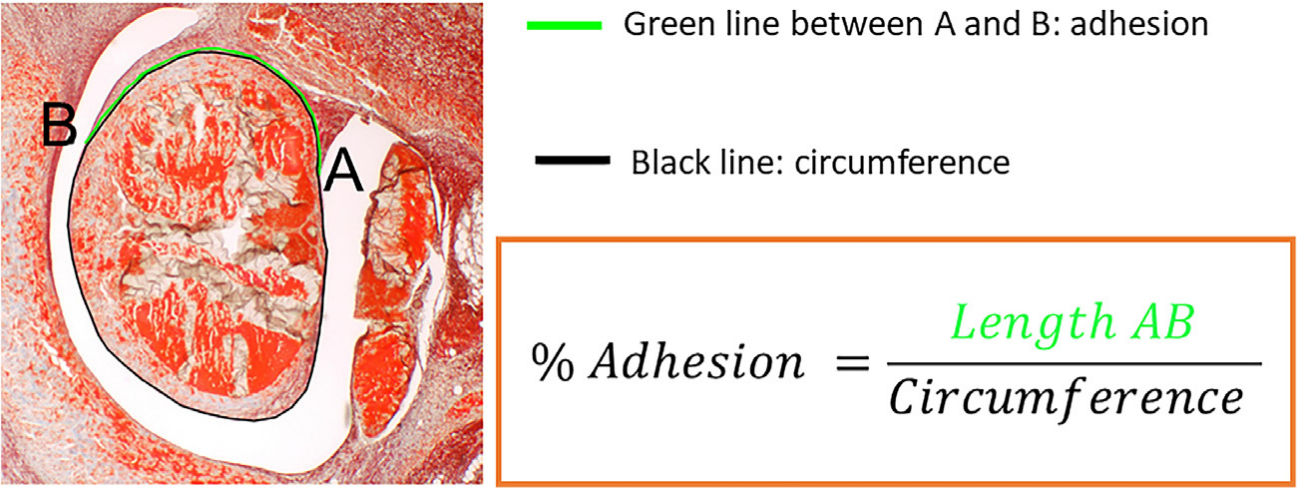
Assessment of percentage of adhesion formation. According to Tan et al. [8], the percentage of adhesion formation can be assessed in tendon cross sections by measuring the length of adherent tissue and dividing it by the length of the whole circumference.

The tendons were defrosted overnight at 4 °C and then warmed to room temperature before they were dehydrated and embedded in paraffin. Perpendicular to the Achilles tendon and in the wound region, cross sections of 5 µm were made, deparaffinized with xylene and rehydrated prior histological staining with Picrosirius Red (PR). To quantify the adhesion extent, PR-stained sections in five sequential cross sections separated by approximately 2.0 mm as described by Tan et al. [8] were analyzed at 8 × magnification with a Leica EZ4D microscope (Switzerland). The calculation of the percentage of adhesion was based on the length of the contact region of the tendon with the surrounding tissue divided by the whole tendon perimeter (Fig. 3), according to a protocol reported previously [8]. These lengths were determined by Synedra view software (version 22.0.0.12)

## Limitations

The presented data of adhesion formation are limited to one time point post-operation, i.e. to 3 weeks. It would be interesting to know whether the prominent anti-adhesive effect of the bi-layered tube would remain over longer periods of time.

## Ethics Statement

We confirm that our experiments complied with the ARRIVE guidelines and were carried out in accordance with the EU Directive 2010/63/EU for animal experiments. Ethical approval for the experiments was achieved from the veterinary office of Zurich, Switzerland (reference numbers ZH 080/2021; 33530).

## CRediT authorship contribution statement

**Iris Miescher:** Conceptualization, Methodology, Formal analysis, Investigation, Data curation, Writing – original draft, Writing – review & editing. **Nicola Schaffner:** Formal analysis, Investigation, Data curation. **Julia Rieber:** Formal analysis, Investigation, Data curation. **Gabriella Meier Buergisser:** Methodology, Formal analysis, Investigation, Data curation. **Esteban Ongini:** Conceptualization, Methodology, Formal analysis, Investigation, Data curation, Writing – review & editing. **Yao Yang:** Methodology, Investigation, Data curation, Writing – review & editing. **Athanasios Milionis:** Methodology, Investigation, Data curation, Writing – review & editing. **Viola Vogel:** Writing – review & editing, Supervision. **Jess G. Snedeker:** Writing – review & editing, Supervision. **Maurizio Calcagni:** Writing – review & editing, Supervision. **Johanna Buschmann:** Conceptualization, Resources, Data curation, Writing – review & editing, Supervision, Project administration, Funding acquisition.

## Data Availability

Mendeley DataFTIR data (Original data). Mendeley DataFiber thickness data (Original data). Mendeley DataPore size data (Original data). Mendeley DataDSC data (Original data). Mendeley DataWCA data (Original data). Mendeley DataMechanicalTests.xlsx (Original data). Mendeley DataInVivo_Adhesion.xlsx (Original data).
